# Impact of Ascites on Morbidity and Length of Hospital Stay: A Large Retrospective Study from a Tertiary Referral Center

**DOI:** 10.3390/medicina62040751

**Published:** 2026-04-14

**Authors:** Ion Daniel Baboi, Maria Nedelcu, Lavinia Alice Bălăceanu, Ioana Valeria Grigorescu, Ion Dina

**Affiliations:** 1 Department of Medical Semiology, “Carol Davila” University of Medicine and Pharmacy, 020021 Bucharest, Romania; daniel.baboi@umfcd.ro (I.D.B.); ioana-valeria.grigorescu@rez.umfcd.ro (I.V.G.); ion.dina@umfcd.ro (I.D.); 2 Gastroenterology Department, “Sf. Ioan” Clinical Emergency Hospital, 042122 Bucharest, Romania; 3 Internal Medicine Department, “Sf. Ioan” Clinical Emergency Hospital, 042122 Bucharest, Romania

**Keywords:** ascites, cirrhosis, Romania, length of stay, ICD10, administrative data, patient age, readmissions, health services research

## Abstract

*Background and Objectives*: Ascites is associated with substantial symptom burden and increased healthcare utilization, and it is observed in patients with advanced disease across multiple etiologies. However, because ascites is a clinical sign rather than a diagnosis category, it can be challenging to study using routine health reporting. In routinely collected hospital administrative data, ascites is commonly captured using International Classification of Diseases, Tenth Revision (ICD-10) code R18, an etiologically non-specific classification whose outcome implications are less documented. We aimed to evaluate the incremental association of R18-coded ascites with length of stay (LOS), readmission burden, and in-hospital mortality in the Gastroenterology and Internal Medicine inpatient department, beyond comorbidity burden and other coded decompensation proxies. *Materials and Methods*: We conducted a single-center retrospective study using routinely collected administrative discharge data from adult inpatient admissions (2015–2023) in the Gastroenterology and Internal Medicine department of a Romanian tertiary-care hospital. Admissions were classified by the presence of ICD-10 R18-coded ascites. Outcomes were LOS, readmission burden (count of subsequent admissions), and in-hospital mortality. Multivariable models adjusted for age, sex, and comorbidity burden (Charlson Comorbidity Index), with additional models incorporating ICD-10-derived decompensation proxies to assess overlap in administrative severity signal. LOS was further examined within Charlson strata to evaluate incremental stratification. *Results*: Coded ascites was associated with higher hospital burden, including longer LOS and greater readmission burden, and with higher in-hospital mortality in partially adjusted models. Within each CCI stratum, LOS remained higher among admissions with R18-coded ascites, supporting incremental stratification beyond comorbidity alone. Furthermore, mobility impairment was an important predictor of LOS. Age-stratified analyses suggested a high-burden phenotype among younger patients and infrequent R18 coding among the very elderly in this cohort. *Conclusions*: These findings support the potential utility of R18-coded ascites as a pragmatic administrative marker for risk adjustment and service planning.

## 1. Introduction

Ascites is an abnormal accumulation of fluid in the peritoneal cavity, frequently due to liver damage (hepatic cirrhosis complicated with portal hypertension) [1,2]. Independent of the underlying cause, ascites can increase inpatient management complexity through the need for specific treatments (including paracentesis in selected cases), the potential for infectious complications, and significant symptom burden. In a 2021 study that validated the Chronic Liver Disease Questionnaire in the Romanian population, ascites was associated with a significant impact on the quality of life of the patients (more so than variceal bleeding or hepatocellular carcinoma) [3].

Electronic hospital datasets are frequently used for service evaluation and benchmarking, although they typically require substantial processing before they become research-ready [4,5]. There is a growing body of literature that aims to validate the identification of cohorts using electronic medical datasets and improve disease identification and classification through this resource [6,7]. However, ascites is a clinical sign rather than a diagnosis category, and it is not consistently represented as a standalone condition in major national and global reporting frameworks; consequently, estimates often rely on primary studies rather than routine health reporting [8,9]. Furthermore, in administrative research, ascites-related work has therefore commonly focused on the validity of coding, whereas outcomes associated with ascites coding have been comparatively less emphasized [10,11].

The aim of this study was to evaluate the incremental association of ICD-10 R18-coded ascites with length of stay (LOS), readmission burden, and in-hospital mortality in routinely collected inpatient data from Gastroenterology and Internal Medicine services, beyond comorbidity burden and other coded decompensation proxies, and to provide a descriptive reference from a Romanian tertiary-care setting. This information can be used to calibrate expectations regarding outcomes associated with administratively defined diagnoses rather than clinically adjudicated phenotypes, acknowledging that administrative coding does not map one-to-one onto bedside clinical assessments.

In routinely collected hospital data, ascites is typically captured using ICD-10 code R18, which is etiologically non-specific and may reflect ascites from multiple causes. The proportion of liver-related ascites among R18-coded admissions is influenced by local case-mix and coding practice; in Gastroenterology-led inpatient care, it is expected to be predominantly liver-related, while in other settings liver-disease could be underrepresented [12]. Accordingly, evaluating outcomes associated with R18-coded ascites as an administrative phenotype is relevant for administrative surveillance and planning.

In lower resource settings, simple administrative markers of severity could be useful for planning and evaluation. Romania provides an illustrative context in which detailed clinical registries are limited and liver disease remains an important public health problem [13,14,15]. In parallel, health-system indicators suggest marked heterogeneity in chronic disease burden, functional limitation, and access to care, which supports context-specific studies that can inform service planning and policy. Although being under the European average in 2020 for people over 65 years old diagnosed with multiple chronic conditions, Romania had higher limitations in daily activities and lower life expectancy and healthy life years for this population [16] As of 2022, it was also a country with lower obesity rates and lower 30-day mortality rates for patients with acute myocardial infarction and stroke, that also had one of the higher unmet medical needs in Europe [16]. This variability, partially caused by high differences in resources within the country and, also, among inpatient and outpatient care (with less accessibility to high-quality outpatient care) [16], argues for particularized studies of patient characteristics that can guide appropriate management and policies.

## 2. Materials and Methods

This is a retrospective observational study based on administrative discharge data of patients who presented to the Gastroenterology and Internal Medicine Clinic of St John Emergency Hospital in Bucharest, Romania, between 2015 and 2023. The study aims to evaluate the increase in-hospital burden associated with the presence of an ascites diagnosis and describe the characteristics of patients with ascites in an administrative perspective, that allows for easier evaluations across multiple units with lower levels of resources.

St John Emergency Hospital is a high-resource, 2nd rank hospital (based on the national classification of medical units). This unit can offer higher complexity procedures (such as timely therapeutic endoscopy and partial splenic embolization for hypersplenism due to liver cirrhosis) but is also the unit that covers lower-complexity cases in the area [17]. Gastroenterology and Internal Medicine departments were analyzed together in this study due to the practical overlap of case typologies.

All patients discharged from the Gastroenterology and Internal Medicine ward during the 2015–2023 interval after a continuous admission were included. Ascites was defined as the presence of the ICD10 diagnosis R18 at discharge (either primary or secondary diagnosis). Patients with the R18 diagnosis were included in the group of patients with ascites, while the others were included in the group of patients without ascites.

Validation studies of administrative coding reported that the ICD10 R18 code had a 97–100% specificity and positive predictive value, but with moderate sensitivity (49.4–61.3%), indicating that a substantial proportion of clinically present ascites may not be captured by discharge coding [11,18]; however, many uncoded cases were described as having ascites that was not clinically significant, which may reduce the likelihood of coding at discharge. Studies recommend incorporating the use of diuretics for better identification of patients with ascites [10]—however, this information was not available in our administrative dataset. For the present analysis, these properties imply that the “ascites-coded” group is likely to represent true, clinically documented/coded ascites, whereas the comparison group may include patients with uncoded ascites. This exposure misclassification would be expected to attenuate associations, as an under-coding-driven shift towards the null. Consequently, our estimates should be interpreted as the association between administratively coded ascites and administratively captured outcomes, acknowledging that the code may preferentially capture clinically salient cases and may not reflect all patients with ascites. Moreover, ICD-10 R18 is an etiologically non-specific ascites code. Although combining R18 with liver disease codes improves the positive predictive value for liver-related ascites in validation studies, the proportion of cirrhosis-related ascites among R18-coded admissions varies by case-mix and coding practice [12]. Although we expected a higher proportion of cirrhosis-related ascites in our patient population (due to being a Gastroenterology and Internal Medicine department), we aimed to assess outcomes associated with R18-coded ascites as an administrative phenotype in this type of setting, without restricting to defined subgroups.

We collected data from the electronic system of the hospital (age at admission, gender, number of admissions, length of hospital stay (LOS), and ICD10 diagnoses—both primary and secondary). Individual case files were not read for this part of the study. All administrative records were available to the investigators, who de-identified the records and structured the administrative reports into patient-based and admission-based tables, transforming the data to fit the statistical methods that would be used. For the patient-based analysis, all admissions in the Gastroenterology and Internal Medicine department in the 2015–2023 interval were used for counting the number of ICD10 diagnoses, evaluating the severity of comorbidities, and counting readmissions.

For evaluating the overall severity of the cases based on administrative data (ICD 10 coding), we used the Charlson Comorbidity index (CCI) [19,20], calculated patient-wise in the 2015–2023 interval. If a particular code was present during this interval for a patient, it was introduced in the score. To avoid duplication, hierarchical rules were introduced for some of the codes. For example, if a patient had both a code for metastatic neoplastic disease and a code for non-metastatic cancer, only the metastatic code would be considered. CCI was calculated on the patient level, gathering all ICD10 codes from all the admissions recorded in the 2015–2023 interval. This approach allows for an estimated of the cumulative comorbidity during the entire interval and is not restricted to codes present at a particular admission (especially given the fact that acute diagnoses tend to be included far more often than diagnoses that are not immediately relevant to a particular admission). Therefore, in this study, the CCI should be interpreted as a severity proxy that is temporally aggregated and not as a prospective risk score.

A score of decompensation (DS) was used for further analyzing patients with ascites, which was a sum of coded complications associated with decompensated disease, such as varices, digestive bleeding, hepatic encephalopathy, hepatic insufficiency, portal hypertension, hepatorenal syndrome, sepsis, renal insufficiency, hepatocellular carcinoma. Each complication was recorded as a binary (0/1) and summed. The DS was used for a severity-proxy sensitive analysis. The detailed rules for the CCI and the DS are presented in Appendix A.1. The cases were further analyzed for other clusters of ICD10 diagnoses, which are detailed in Appendix A.2.

The main outcomes analyzed in the study were the length of hospitalization (LOS), number and odds of readmission, and mortality. We also evaluated the characteristics of the patients stratified by age.

The statistical analysis was performed using IBM SPSS Statistics 26 and Microsoft Excel. The statistical significance was established at *p* < 0.05. The paper is structured using the STROBE/RECORD 2015 checklist [21].

For the comparison of the outcomes between the group of patients with ascites and patients without ascites, we used two models adjusted for the severity of the cases, using the CCI and the DS. Model A is adjusted for age, gender, and CCI, for a first comparison between the groups. Model B added the ICD-10–derived decompensation proxy score to Model A. Because DS components may reflect downstream severity markers that co-occur with clinically salient ascites, it is interpreted as a sensitivity analysis including a decompensation proxy.

The B model must be interpreted as a stability analysis that evaluated the superposition of the administrative signals, and not as a causally adjusted model. The components of the DS include decompensation events that could co-occur with ascites. Adjusting for DS in the model used for the presence of R18 is a super-adjustment, that is expected to attenuate the association with morbidity outcomes, and is not used to control independent confusion factors. We use Model A (adjusted for age, gender, CCI) as the main analytic model, and we only include the B model to address the degree of superposition between ascites (R18) and other decompensation proxies.

LOS was modeled through GLM Gamma with log link, reporting exp(*β*) as a multiplicative effect on LOS associated with the presence of ascites. The smallest LOS value was stored in the administrative records as one day. Readmissions were evaluated for patients discharged alive using a negative binomial model. Readmissions were cumulated for the whole interval, and the IRR must be interpreted as burden during the observed period, since they are not time-standardized and influenced by differing lengths of follow-up, depending on year of first admission. Odds of readmission during the interval and mortality were modeled through logistic regression, reporting OR. For the estimation of survival, 634 patients with ascites were analyzed longitudinally, reading individual patient records.

## 3. Results

**Figure 1:**
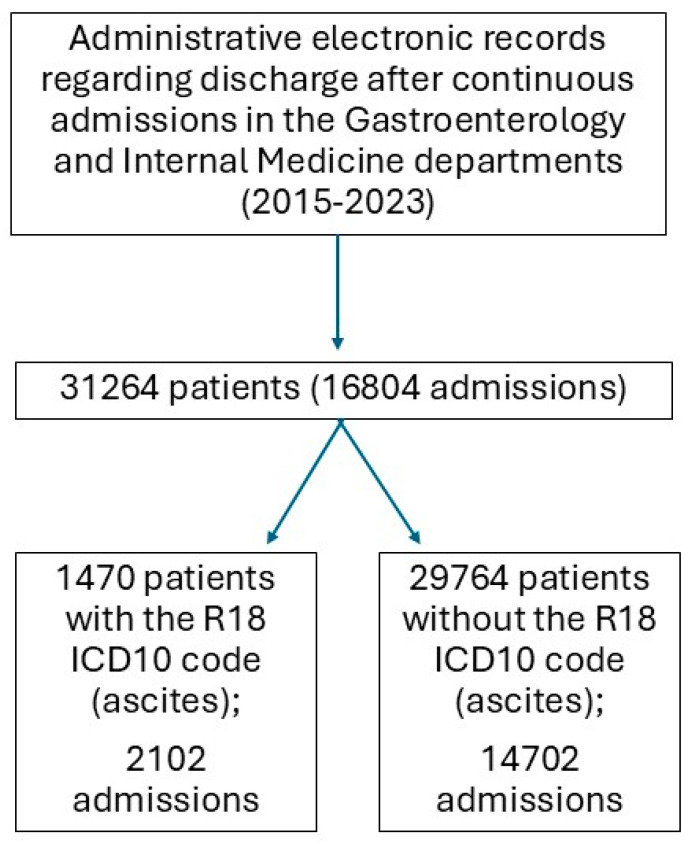
Flow diagram of the study population selection.

There were 13,264 patients in total (16,804 admissions), among which 1470 patients had a diagnosis of ascites (R18) (2102 admissions)—Figure 1. Although the overall gender distribution was quite even, the patients with ascites were predominantly males (66.9%, *n* = 984, χ^2^ = 210.45, df = 1, *p* < 0.001). 63% of the patients had an ascites diagnosis combined with a cirrhosis diagnosis, 16% with both cirrhosis and malignant disease and 12.5% with malignant disease.

The general characteristics of the groups of patients with and without ascites are presented in Table 1 and Table 2.

### 3.1. Age at First Admission

Ascites was more frequent for the 50–59 and 60–69-year-old age interval (17% and 14%) than for the 20–29 (2%), 30–39 (10%), 70–79 (8%) and over 80 (5%). In comparison, in the non-ascites group, the age distribution is more balanced across age-groups. The probability of ascites increases with age (χ^2^ = 228.47, df = 3, *p* < 0.001), followed by a decrease in very elderly patients. Patients with ascites were younger, but had a higher CCI and DS than patients without ascites.

**Figure 2:**
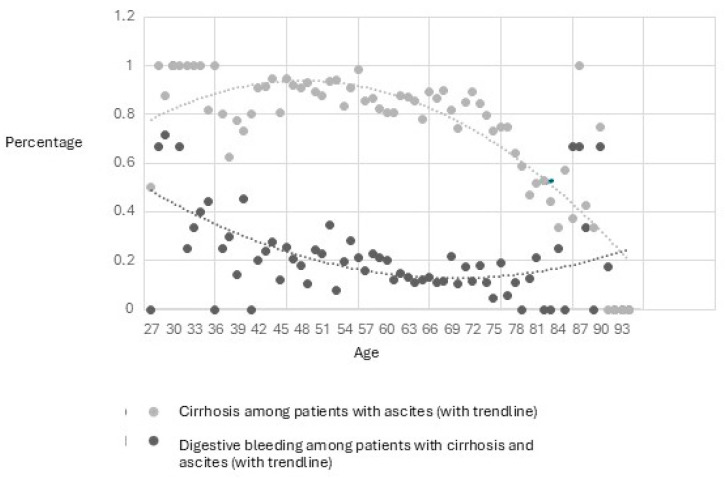
Proportional differences in cirrhosis frequency among patients with ascites of different ages (gray dots). For patients with cirrhosis and ascites, the black dots show the proportion of cases with gastrointestinal bleeding (black dots).

Very elderly patients were rare in the ascites group, and the etiology of ascites was less frequently cirrhosis and more frequently cardiac. All cases of ascites above 85 years old were individually verified for clusters of cardiac diagnoses and liver diagnoses. Out of the 58 patients who were 95 years or older, only two had ascites, and both had a cardiovascular etiology (neither had hepatic, splenic or malignant ICD10 diagnoses, but they had multiple cardiac comorbidities, including cardiac insufficiency). In the 90–95 age group, there were nine patients with ascites (299 patients in total), and only one of them did not have a cardiac insufficiency diagnosis. This patient was a 90-year-old female with a malignant tumor of the liver, hypersplenism, hypertension, ischemic cardiomyopathy and atrial fibrillation. Both her and the other two patients in this age group that had liver disease (and a possible mixed etiology of ascites—cardiac and hepatic) presented with digestive bleeding through varices (*n* = 2) or hemorrhagic gastritis (*n* = 1) and one of them died during the hospitalization. In the 85–89 age group there were 879 patients in total and 35 with ascites, out of which 15 had cirrhosis diagnoses, and the others had only cardiac insufficiency diagnoses. For patients younger than 75, the percentage of patients with cirrhosis diagnoses increased to 80–100%. Figure 2 shows the proportional differences in cirrhosis frequency among patients with ascites in relation to the age of the patients.

Moreover, the frequency of digestive bleeding (ICD10 codes used for digestive bleeding: I85.0, K25.0, K26.0, K26.2, K27.0, K28.0, K92.0, K92.1, K92.2) was higher for younger patients with cirrhosis and ascites: 30.2% in those aged ≤ 45 years, significantly higher compared with 16.7% in 46–75 years and 13.8% in ≥76 years (χ^2^ test *p* < 0.001), which can also be seen in Figure 2. In our cohort of all admitted patients in the gastroenterology and internal medicine ward (regardless of the disease), digestive bleeding was more frequent under 60 years of age (11.9%), than in the 61–79 and over 80 categories (9.5% and 9.2%, χ^2^ test, *p* < 0.001).

Kruskal–Wallis analysis followed by the Dunn–Bonferroni analysis showed significant differences between age groups (under 40, 40–60, 60–80, over 80) for number of admissions, number of ICD10 diagnoses and LOS. Patients under 40 years old with ascites had more admissions than the other groups and longer hospitalizations, associated with more aggressive causes of ascites in this population (although the number of diagnoses was lower).

### 3.2. Readmissions and Length of Hospitalization

Coded ascites was associated with a higher readmission count in Model A (IRR = 2.249; *p* < 0.001). After addition of DS (Model B), the association attenuated but remained statistically significant (IRR = 1.375; *p* = 0.0076), consistent with DS capturing only part of the administrative risk signal carried by ascites coding. In the binary readmission model (any readmission vs. none), R18-coded ascites was associated with higher odds of readmission in Model A (OR = 2.126), which remained true, although less prominent, in Model B (OR = 1.342).

We also evaluated readmissions at 30 days and 90 days. Patients with ascites were 2.6 (CI 2.1–3.22) times more likely than patients without ascites to be readmitted at 30 days and 2.55 (CI 2.06–3.14) times more likely to be readmitted at 90 days.

A total of 59.09% of patients with ascites were admitted for 7–29 days and 0.95% for more than 30 days (compared to 43.35% and 0.71% in the overall population of inpatients). The relatively high number of longer than 7 days admissions in both groups could be contextualized by an observation regarding the difficulty of the cases and the relatively high number of patients with severe chronic disease: 7281 out of 16,804 admissions (43.3%) involved patients that were either older than 85 years old or had significant comorbid diagnosis categories (sepsis, dementia, malnutrition/cachexia, severe mobility impairment or renal, stroke, respiratory or severe cardiovascular disease), and 15% of admissions were associated with a ICD10 code F00-F99 (Mental, Behavioral and Neurodevelopmental Disorders).

**Figure 3:**
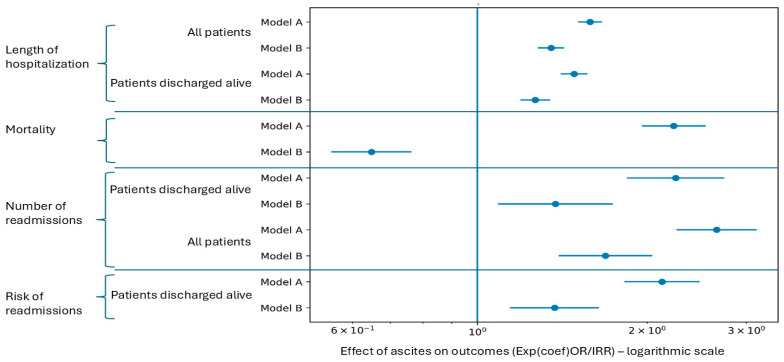
The effect of ascites on four outcomes (LOS, mortality, number of readmissions and odds of readmission). Model A and Model B are two levels of adjustment: Model A adjusts for age, gender and Charlson Comorbidity Index, while Model B additionally includes a decompensation proxy score based on ICD-10 markers (severity-proxy sensitivity analysis).

In Model A, ascites was associated with a higher LOS (exp(β) = 1.585; CI 95% 1.509–1.665; *p* < 0.001). Each point of CCI increased LOS with an average of 5.3%. In Model B, the lengthening remained significant, but the difference was smaller (exp(β) = 1.351; CI 95% 1.279–1.427; *p* < 0.001)—Figure 3. The DS was an important predictor of LOS, as expected (exp(β) = 1.090 per point; *p* < 0.001).

Within each CCI stratum, admissions with ascites had higher mean and 75th-percentile LOS than admissions without an ascites code, indicating that ascites provides incremental administrative risk stratification beyond comorbidity alone. (Table 3). This remained true when data was further categorized based on the DS, the presence of ascites consistently associating longer average LOS across categories.

In order to test the persistence of the negative effect of ascites in the group of patients with neoplastic conditions, we used two methods: first, we compared the mean LOS in admissions with or without ascites, separated for the group of patients without a diagnosis of cancer and for the group of patients with a diagnosis of cancer (by two separated independent samples T tests). The effect was slightly smaller for patients with cancer, but remained significant (mean difference −2.8 days for patients without ascites [CI −3.93; −1.66], *p* < 0.0001 in the group of neoplastic patients and mean difference −6.47 days for patients without ascites [CI −7.36; −5.57] for patients without cancer, *p* < 0.0001). Then, to verify, we grouped the patients based on the median LOS (staying less than 7 days or more than 8 days in the hospital) and confirmed through the χ^2^ test that for both patients with or without neoplasms ascites was associated with longer LOS (patients with neoplasms: χ^2^ = 19.229, *p* < 0.0001; patients without neoplasms: χ^2^ = 202.977, *p* < 0.0001).

Another observation is that the presence of ascites could be associated with a reduction or even disappearance of the association of various comorbidities with an increase in the LOS. The presence of severe malnutrition was associated with an increase in average LOS for admissions without ascites (1.17 days (0.56–1.77), *p* < 0.001) but did not associate a significant increase for admissions with ascites (Those differences are based on average comparisons and are unadjusted). Similarly, the presence of dementia was associated with an increase in LOS for admissions without ascites (1.88 (1.38–2.38), *p* < 0.001) but did not associate a significant increase for admissions with ascites. This pattern remained true for severe cardiovascular comorbidities, obesity, and cerebrovascular infarction. The presence of selected severe respiratory comorbidities was associated with a 3.08 day (2.86–3.29) increase in LOS for admissions without ascites (*p* < 0.001) and no significant increase for all patients with ascites, but with a smaller, 1.51 days increase (0.46–2.64) (*p* = 0.006) for admissions with ascites discharged alive. However, the presence of severe mobility impairment associated the highest increase in LOS: 6.33 days (5.34–7.42) for admissions without ascites (*p* < 0.001), and 3.57 days (1.03–1.51) in patients with ascites (*p* = 0.001). For patients with ascites discharged alive, the increase was higher (5.07 days (1.78–8.35), *p* = 0.004).

### 3.3. Comorbidities

Patients with ascites had more ICD10 diagnoses and a higher CCI. Table 4 shows the mortality of the most frequent ICD10 diagnoses in the population of patients with ascites. Mortality was more than 30% higher for the subgroup of patients with ascites than for the general patient population for sepsis, hematemesis, acute respiratory failure and double or more than double for viral pneumonia, acute hemorrhagic gastritis and acute pancreatitis.

### 3.4. Mortality

Mortality was 33.5% among patients with ascites and 16% among patients without ascites. In Model A, ascites was associated with a higher odd of death (OR = 2.231; CI 95% 1.958–2.541; *p* < 0.001). In Model B, the decompensation score (DS) was strongly associated with in-hospital mortality (OR = 2.246 per point; *p* < 0.001), and the association between R18-coded ascites and mortality was substantially attenuated and no longer statistically significant. This pattern suggests that the excess mortality observed in Model A is largely captured by coded decompensation severity proxies included in the DS. Model B results are interpreted as describing overlap between ascites coding and coded decompensation proxies, rather than establishing the absence of an effect of ascites per se.

To address the negative effect of ascites regarding LOS and mortality in patients with neoplasia, we performed the χ^2^ test and found that the presence of ascites was associated with a significantly higher mortality rate both for patients with neoplasms and patients without neoplasms (patients with neoplasms: χ^2^ = 8.201, *p* = 0.004; patients without neoplasms: χ^2^ = 267.099, *p* < 0.0001).

**Figure 4:**
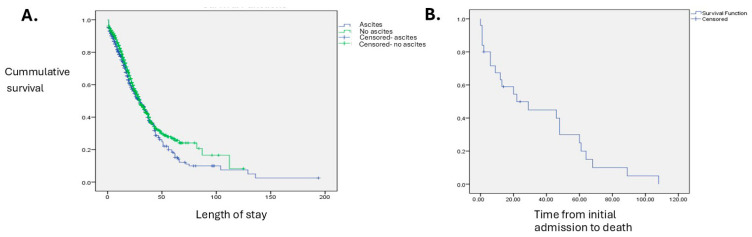
(**A**) Kaplan–Meier curves using LOS as the time scale and censoring at discharge. Curves diverge at longer LOS, with lower cumulative in-hospital survival among admissions with R18-coded ascites. (**B**) Survival analysis for patients with ascites, using time from the first admission to death in the hospital (days) as the time variable.

For assessing the survival for a particular hospitalization event in both groups, we used Kaplan–Meier survival analysis. When using length of stay as the time scale and censoring at discharge, survival curves diverged at longer lengths of stay, with lower late in-hospital survival among admissions with R18-coded ascites. This pattern should be interpreted descriptively because discharge alive is a competing event and LOS is influenced by clinical course and discharge decisions (Figure 4A).

For a subgroup of 634 patients that were analyzed more in depth (using individual patient files), we evaluated the overall survival of patients with ascites (in-hospital death was the event and the duration from the initial admission to death was the time variable). The mean survival time was 35 months (Ci 22–48), and the median of survival (the moment half the patients were still alive) was 22 months (Figure 4B). About a quarter of the patients survived for fewer than 6 months, and another quarter for more than 5 years, depending on the etiology of ascites and the response to treatment. The curve shows higher early hazard (during the first 2–3 years) with a less steep decline among longer-term survivors. The survival analysis results were similar to other studies on patients with ascites [22,23].

## 4. Discussion

The presence of an ICD-10 R18 ascites code was associated with higher readmission counts, longer LOS and higher in-hospital mortality after adjustment for age, sex, and CCI. Those findings remained valid for the group of patients with neoplasia. After additional inclusion of an ICD-10-derived decompensation proxy score, the associations attenuated—consistent with shared severity signal between ascites coding and other coded decompensation markers—while the increases in readmissions and LOS remained statistically significant. Stratified analyses further showed that, within each CCI stratum, LOS metrics were consistently higher among admissions with R18-coded ascites, suggesting that it captures additional administrative severity/burden not fully accounted for by comorbidity level. Those findings are consistent with the literature: in one review of patients with cirrhosis, ascites caused the highest rate of readmissions compared to gastrointestinal bleeding and hepatic encephalopathy [24]. Another study found that most of the admissions of patients with ascites are directly related to its management, and almost half of them are unplanned [25]. The effect of the R18 code, however, is interesting to report separately, since it is known that coding is more likely in salient cases and is dependent on the impact of the condition in the broader picture of the patient condition [11,18].

Validation studies have reported high specificity and positive predictive value for R18, albeit with moderate sensitivity, suggesting that R18 preferentially captures clinically salient ascites and should be interpreted primarily as a rule-in marker rather than a comprehensive ascertainment tool [11,18]. In this context, incorporating R18 into administrative risk models may improve stratification of expected resource use (e.g., LOS and readmission burden) beyond comorbidity indices alone.

More broadly, administrative identification of liver disease often relies on code bundles to improve sensitivity and to capture decompensation events; within such frameworks, R18 may contribute both to case ascertainment and to severity stratification [26]. This may be particularly relevant considering that some other decompensation indicators tend to be inconsistently coded (e.g., hepatic encephalopathy), limiting their utility as standalone administrative markers [27]. Where diagnosis coding is available at, or near, admission, R18 could potentially support earlier risk stratification. However, future studies are needed to confirm those retrospective findings and validate them in other centers.

Collectively, these findings support the use of ICD-10 R18-coded ascites as an administrative marker of a subgroup of patients with higher expected resource use in Gastroenterology and Internal Medicine settings, when physiological severity measures are unavailable at scale. Similar administrative markers are used in practice for case-mix adjustment and, more cautiously, for capacity planning and for the identification of patients that might benefit from more tailored interventions [28,29]. In a meta-analysis by Mao et.al., more than half of the prediction models for readmissions were based on retrospective administrative data [29]. Scores such as the Hospital Frailty Risk Score, for example, are explicitly built from ICD-10 codes to flag a high-risk group and are shown to relate to outcomes predicting a higher risk of longer LOS or higher readmission count could be used to identify patients in which a transitional care intervention might be needed. In this context, if validated locally, R18-coded ascites could be used to flag patients who may require earlier discharge planning, closer coordination of follow-up, and anticipatory allocation of ascites-related resources. In particular, prior literature suggests that timely diagnostic paracentesis in hospitalized patients with cirrhosis and ascites is associated with lower mortality and shorter hospital stay, and that dedicated outpatient/day-case paracentesis services may reduce admissions, emergency department utilization, inpatient bed days, and costs [30]. Furthermore, recognition of the higher-risk context in which ascites often occurs, particularly in decompensated disease, may support earlier goals-of-care and advance care planning discussions with patients and families when clinically appropriate. These applications were not tested in the present study and should therefore be interpreted as potential service-planning implications to be evaluated in future prospective work.

Our data also suggested an attenuated increase on LOS by several patient-level predictors in admissions with ascites (such as dementia, malnutrition, respiratory disease). However, there was a marked association of mobility impairment with high LOS that was significant for both groups. Although these patterns may partly reflect differences in sample size or coding practices, they support the intuition that, in patients with a decompensation of a severe chronic condition (such as ascites), the severity of the decompensation can outweigh other social or practical determinants of LOS. The pattern of loss of significance and the high association of mobility impairment with LOS for both groups may be informative for the development of future prospective studies regarding the growing interest in how frailty and multimorbidity influence patient prognosis and hospital burden [28,31,32]. These subgroup patterns are exploratory and should be interpreted as hypothesis-generating, as differential coding intensity, severity selection and sample size could contribute to apparent attenuation.

We observed two administrative patterns regarding the age of the patients with ascites: on one hand, ascites was very rare in the elderly patients and was mostly associated with a cardiac etiology in this subgroup. On the other hand, younger patients with ascites and cirrhosis had a higher frequency of digestive bleeding and more severe outcomes. These age-stratified patterns may reflect a combination of etiologic heterogeneity, survivors selection, and patterns of coding. The prior literature supports that cirrhosis phenotype and complication profiles vary meaningfully by age, which provides context for the administrative patterns observed here [33,34,35,36].

The average LOS of admissions without ascites in the internal medicine and gastrointestinal ward in our unit (8.3) was longer than the national average LOS reported for the year 2021 (7.7) and longer than the one reported for digestive system diseases (5.6) [37,38]; possibly, this reflected the overlap of gastrointestinal and internal medicine cases and referrals of more complex patients from lower-resource units. Using the NHS categorization of LOS, 44.06% of all patients were stranded (admitted for more than 7 days) or super stranded (admitted for more than 30 days), a number that increased to 57.04% for patients with ascites. Moreover, the average LOS of patients with ascites was higher than in other studies including patients with ascites (13.9 compared to 4.7 [39], 6.7–12.2 [40], or 10.28 [41]. Direct LOS comparisons across studies should be interpreted cautiously because cohorts differ in ascites definitions (clinical ascites vs. discharge coding) and etiologic mix, and the higher LOS could be associated with the increased severity of coded ascites. However, the overall tendency of longer LOS could be further contextualized by the following discussion about local healthcare characteristics.

Although Romania has reduced LOS and expanded day-care discharges in recent years, inpatient care still absorbs a high share of healthcare expenditure compared with other European countries [37,38]. A 2021 study in 10 public hospitals found that over half of pediatric and obstetric hospitalizations were unnecessary, with frequent unnecessary prolongation of stays, largely due to limited outpatient diagnostics and concerns about families’ ability to provide safe care at home [42], a pattern likely mirrored by other specialties. Moreover, acute, chronic and palliative care is not clearly delineated, with inpatient care in emergency tertiary hospitals is overused due to limited access to rehabilitation, long term and palliative services [43,44]. In 2017, more than 75% of patients in need of palliative care were managed at home almost solely by family members, and there were people who wished to die at home and were unable to, due to the inability or unwillingness of the family to provide care [45]. LOS is also affected by the admission of “social cases”, estimated to account for up to 10% of admissions in a Romanian 2017 study [42,46,47,48]. Initiatives such as the Pal-Plan started in 2023 by the Ministry of Health are starting to improve the issues [49]. In line with the Romanian Ministry of Health’s Order number 3514 from October 2023, liver insufficiency in the context of Child B or C cirrhosis, with a MELD score higher than 14, or with refractory ascites, advanced neoplastic disease and advanced cardiac insufficiency are all included among the pathologies eligible for palliative care services.

### 4.1. Further Research

Further research could validate the role of the R18 ICD10 code as a marker of severity in administrative analyses in different settings and evaluate the role of classical LOS modifiers for patients with ascites. Moreover, because ascites is not reported as a separate disease entity, much of the insight into its epidemiology comes from the literature rather than routine health reports. Bibliometric analyses can map trends and research gaps, and several such studies exist in liver disease, including cirrhosis and digestive bleeding [50,51,52], but we found none focused specifically on ascites.

We underline the paucity of performance studies on cirrhosis and ascites care—especially in Romanian hospitals—and the need to calibrate biomarkers and clinical assessments for this population [53]. Also, due to its prevalence and morbidity, ascites can serve as a useful window on hospital efficiency and costs, using tools such as data envelopment analysis (DEA) and economic burden studies [46,50].

### 4.2. Limitations

This study has limitations inherent to the use of routinely collected, single-center administrative data that were not generated for research purposes. Ascites status and other clinical states were ascertained exclusively from ICD-10 discharge diagnoses without clinical validation, introducing potential misclassification due to undercoding, overcoding, and variability in documentation and coding practices. Because validation studies of ICD-10 R18 generally report high specificity and positive predictive value but moderate sensitivity, misclassification is likely to be asymmetric: the non-R18 comparison group may include uncoded, less clinically salient ascites, which would tend to attenuate observed contrasts between groups. The administrative dataset also lacked granular clinical severity and process-of-care measures (e.g., MELD/Child–Pugh scores, patient-reported quality of life, sarcopenia/frailty markers, inflammatory indices, etiology-specific biomarkers, and care-process factors such as timing of paracentesis), limiting adjustment for residual confounding by severity [30,51,52,54,55,56,57,58].

Another limitation is that the LOS and mortality were evaluated on the admission level—therefore, if part of the patients contribute with multiple admissions, the standard errors could be slightly underestimated due to intra-patient correlations, and the results must be interpreted in this light. However, the analysis of readmissions, that might be one of the most influenced by this effect, remained valid for both patient-wise and admission-wise analyses.

Moreover, since CCI was aggregated for the whole time-interval for each patient, for patients with a longer window of observation, more diagnoses might be included (bias look-ahead), and we recognize this as a limitation of the study. The study period also overlaps with the COVID-19 pandemic, and delayed healthcare-seeking may have contributed to more advanced presentations within the cohort. [59]. Moreover, a 2024 study on hepatocellular carcinoma diagnoses in Romania showed that a large proportion of patients were diagnosed through a decompensatory event and up to a quarter of the patients were diagnosed with terminal disease [60]. Additionally, late presentations of non-hepatic malignancy can occur in local practice [61]. Taken together, these observations suggest that administrative “complexity” indicators (such as comorbidity counts) may disproportionately reflect decompensated, high-acuity states in this setting, which could influence effect sizes and limit direct comparability with cohorts from systems with earlier access to specialist and outpatient care.

Despite these limitations, the present study provides a pragmatic administrative description of admissions with R18-coded ascites and quantifies its incremental association with hospital-burden outcomes in routine inpatient data. The consistency of associations across models and comorbidity strata suggests that R18 coding contains severity signal not fully captured by comorbidity indices alone, supporting its potential utility for administrative risk adjustment and service planning when physiological severity measures are unavailable at scale.

## 5. Conclusions

This ICD-10-based single-center study compared admissions with R18-coded ascites to other inpatient admissions and found consistent associations with higher hospital burden, including longer length of stay (LOS), and higher readmission burden after adjustment for comorbidity and decompensation proxies, alongside higher mortality and a higher coded comorbidity burden. These findings support the potential utility of R18-coded ascites as a pragmatic administrative marker for risk adjustment and service planning when physiological severity measures are unavailable at scale. Age-stratified analyses suggested that R18-coded ascites was uncommon among the very elderly in this cohort and, when present, was more frequently accompanied by non-hepatic comorbidity patterns (e.g., cardiac diagnoses), whereas younger patients with R18-coded ascites exhibited a high-burden phenotype characterized by longer LOS, more readmissions, and more frequent gastrointestinal bleeding. Several common LOS-prolonging factors showed attenuated associations within the ascites-coded subgroup, whereas mobility impairment remained strongly associated with LOS.

## Figures and Tables

**Table 1:** The general characteristics of the groups of patients with and without ascites.

	Presence of Ascites	Mean	Median; IQR	Range	Outliers and Comments About the Whole Cohort
Age at first admission	Yes	61.9	62; 15	27–97	- 58 patients 95 or older (35 discharged alive) - 4 patients 100 or older (2 discharged alive)
	No	66.1	68; 21	18–101	
Length of hospitalization	Yes	13.9	10; 12	1–82	- 7405 patients (44.06% of all patients) were admitted for 7 or more days - a few outliers stayed for up to 82 days - one extreme outlier discharged alive after 125 days (diagnoses: diverticulosis, anemia, esophagitis, gastritis, pressure ulcers, cachexia and COVID-19 pneumonia)
	No	8.3	6; 6	1–125	
Number of admissions	Yes	1.7	1; 1	1–29	- the patient with 29 readmissions came during the last 4 years of life with hepatorenal insufficiency, hypersplenism and thrombocytopenia
	No	1.2	1; 0	1–13	
Number of ICD10 diagnoses	Yes	15.4	14; 9.2	2–52	
	No	10.6	10; 8	2–57	
Charlson Comorbidity Index (2015–2023)	Yes	4.4	4; 3	0–15	- 18% of all patients had an index of 10 or higher
	No	2.8	2; 3	0–16	

**Table 2:** The number of patients with and without ascites that were assigned to diagnoses categories, based on ICD10 codes: bold categories are part of the Charlson Comorbidity Index. The ICD10 codes included for the other categories were: Severe mobility impairment (L89, S68.2, S78.1, S88.0, S88.9, S98.1, S98.2, S98.4, T13.6, T87.4, T87.5, T87.6) and Severe malnutrition (E43, R64).

Diagnoses	Presence of Ascites	No.
**Myocardial Infarction**	Yes	23
	No	468
**Cardiac Failure**	Yes	276
	No	3320
**Peripheral Vascular Disease**	Yes	125
	No	2050
**Cerebrovascular Disease**	Yes	72
	No	1678
**Pulmonary Disease**	Yes	120
	No	2030
**Rheumatic Disease**	Yes	4
	No	81
**Peptic Ulcer**	Yes	94
	No	727
**Liver Disease (Severe, Mild)**	Yes	1108/186
	No	1008/4104
**Diabetes Mellitus (with/without Complications)**	Yes	222/72
	No	2073/705
**Hemiplegia or Paraplegia**	Yes	2
	No	144
**Renal Disease**	Yes	221
	No	1728
**Neoplasy (with/without Metastases)**	Yes	160/169
	No	795/820
**AIDS**	Yes	3
	No	13
**Dementia**	Yes	28
	No	730
Severe Mobility Impairment	Yes	43
	No	471
Severe Malnutrition	Yes	141
	No	670

**Table 3:** The length of hospitalization (LOS) for different categories of Charlson Comorbidity Indexes (CCI). SD = standard deviation.

CCI	Mean LOS (No Ascites)	SD (No Ascites)	75th Percentile (No Ascites)	Mean LOS (Ascites)	SD (Ascites)	75th Percentile (Ascites)
0	7	6	8	9	4	11
1–4	8	7	10	13	12	17
5–8	10	9	13	16	17	20
9–12	10	10	12	14	15	15

**Table 4:** The mortality of the most frequent ICD10 diagnoses in the population of patients with ascites, listed in a descending order, compared to the mortality of those diagnoses in the overall population of patients. Increasing percentages are highlighted based on a three-color scale (white–yellow–red).

ICD10 Code.	Diagnosis (Sorted by Incidence Among Patients with Ascites)	Mortality (Among Patients with Ascites)	Mortality (Among All Patients with the Diagnosis)
K71.7	Toxic liver disease with cirrhosis and fibrosis of the liver	5.53%	5.90%
K72.1	Chronic hepatic insufficiency	18.14%	20.00%
K74.6	Other cirrhosis of the liver	21.13%	11.62%
K72.0	Acute and subacute liver insufficiency	87.72%	87.25%
J96.0	Acute respiratory failure	38.46%	23.93%
I85.0	Bleeding esophageal varices	4.35%	4.55%
K70.3	Alcoholic cirrhosis of the liver	7.50%	4.84%
G92	Toxic encephalopathy	43.75%	35.29%
I50.0	Congestive cardiac insufficiency	9.38%	7.46%
K92.0	Hematemesis	16.13%	11.83%
C22.9	Malignant tumor of the liver, unspecified	6.45%	5.26%
K85	Acute pancreatitis	18.18%	3.70%
C22.0	Hepatic cell carcinoma	21.05%	14.89%
K29.0	Acute hemorrhagic gastritis	5.56%	1.97%
R40.2	Coma, unspecified	86.67%	78.38%
J12.9	Viral pneumonia, unspecified	20.00%	9.05%
A41.9	Sepsis, unspecified	64.29%	47.33%

## Data Availability

The raw data supporting the conclusions of this article will be made available by the authors on request.

## Abbreviations

The following abbreviations are used in this manuscript:

LOS	Length of stay

## Appendix A

### Appendix A.1

**Comorbidity**	**ICD10 Code**	**Comments**
Myocardial infarction	I21.*, I22.*, I25.2*	
Congestive cardiac failure	I50.*, I11.0, I13.0, I13.2	
Peripheral vascular disease	I70.*, I71.*, I73.1, I73.8, I73.9, I77.1, I79.0, I79.2, K55.1, K55.8, K55.9, Z95.8, Z95.9	
Cerebrovascular disease	I60–I69, G45.*, G46.*	
Dementia	F00–F03, F05.1, G30.*	
Chronic pulmonary disease	J40–J47, J60–J67, I27.8, I27.9, J68.4, J70.1, J70.3	
Rheumatic/Connective tissue disease	M05.*, M06.*, M32–M34, M35.3, M36.0	
Peptic ulcer	K25–K28	
Mild hepatic disease	B18.*, K70.0–K70.3, K70.9, K71.3–K71.5, K71.7, K73.*, K74.*, K76.0, K76.2–K76.4, K76.8–K76.9, Z94.4	If the patient had a Moderately Severe hepatic disease category coding, this category was hierarchically set to 0.
Moderately severe hepatic disease	I85.*, I86.4, I98.2, I98.3, K70.4, K71.1, K72.*, K76.5, K76.6, K76.7	
Diabetes without complications	E10–E14 with subcode 0.0/0.1/0.9	If the patient had a Diabetes with complications coding, this category was hierarchically set to 0.
Diabetes with complications	E10–E14 with subcode 0.2–0.8	
Hemiplegia/paraplegia	G81.*, G82.*, G83.0–G83.4, G04.1, G11.4, G80.1, G80.2, G83.9	
Chronic renal disease	N18.*, N19, N25.0, I12.0, I13.1, N03.2–N03.7, N05.2–N05.7, N07.2–N07.7, N11.0, N14.0–N14.2, N15.0–N15.1, N15.8, N16.0, Z49.0–Z49.2, Z94.0, Z99.2	
Non-metastatic malign neoplasy	C00–C76, C81–C97	If the patient had a Metastatic malign neoplasia coding, this category was hierarchically set to 0.
Metastatic malign neoplasy	C77–C80	
HIV/AIDS	B20–B24	
DECOMPENSATION:		
Esophageal varices with bleeding	I85.0	This and the “Esophageal varices without bleeding” hierarchically organized, so that bleeding would be dominant in an association.
Other varices	I85.*, I86.4, I98.2, I98.3	
Esophageal varices without bleeding	I85.9	
Digestive bleeding	K92.0, K92.1, K92.2	
Encephalopathy, hepatic insufficiency	K72.*, G93.4	
Portal hypertension	K76.6	
Hepatorenal syndrome	K76.7	
Sepsis	A40.*, A41.*	
Acute or chronic renal insufficiency	N17.*, N18.*, N19	
Peritonitis	K65.0, K65.8, K65.9, K65.2	
Primary hepatic cancer	C22.*	
The asterisk indicates inclusion of all subcodes within the respective ICD-10 category (i.e., all codes following the decimal point).		

### Appendix A.2

- Digestive Bleeding (I85.0, K25.0, K26.0, K26.2, K27.0, K28.0, K92.0, K92.1, K92.2)
- Dementia (F00.0*, F00.1*, F00.2*, F00.9*, F01.1, F01.3, F01.8, F01.9, F02.0*, F02.3*, F02.8*, F03, F05.1)
- Severe malnutrition and cachexia (E43, R64)
- Severe mobility impairment (L89, S68.2, S78.1, S88.0, S88.9, S98.1, S98.2, S98.4, T13.6, T87.4, T87.5, T87.6)
- Respiratory Comorbidities (A15.0, A15.1, A15.3, A16.0, A16.2, I26.0, I26.9, I27.0, J10.0, J11.0, J18.0, J18.9, J44.0, J44.1, J81, J84.1, J96.0, J96.1, J96.9)
